# Epidemiology and genetic analysis of *Oestrus ovis* from slaughtered sheep in Sulaymaniyah province, Iraq

**DOI:** 10.2478/jvetres-2025-0019

**Published:** 2025-03-25

**Authors:** Aram Ahmad Mohammed, Taib Ahmed Hama Soor

**Affiliations:** Department of Microbiology, College of Veterinary Medicine, University of Sulaimani, Sulaymaniyah 46001, Iraq; College of Health and Medical Technology, Sulaimani Polytechnic University, Sulaymaniyah 46001, Iraq; Department of Medical Laboratory Analysis, College of Health Sciences, Cihan University-Sulaimaniya, Sulaymaniyah 46001, Iraq

**Keywords:** oestrosis, *COX1* gene, sheep, phylogenesis, Iraq

## Abstract

**Introduction:**

Oestrosis is a type of nasal myiasis that is caused by larvae of flies from the *Oestrus* genus and is a disease of economic significance in small ruminants. The research aimed to investigate the prevalence of oestrosis and detect differences in the *COX1* gene among haplotypes found in Sulaymaniyah, in the Kurdistan region of Iraq.

**Material and Methods:**

The research was conducted in a Sulaymaniyah abattoir from September 2023 to August 2024. The heads of 328 sheep were carefully incised and inspected to record the larvae of *Oestrus ovis*. A conventional polymerase chain reaction and sequencing of the *COX1* gene were used for diagnosis and genetic analysis of *O. ovis*.

**Results:**

The rate of oestrosis was 22.25% and the disease was significantly (P-value < 0.05) higher in imported breeds (26.50%) than the local breed (15.65%), in adults (26.88%) than in young animals (16.19%), in females (27.05%) than in males (17.08%), and in the summer (34.09%) than in other seasons of the year. Comparison of the sequences of the conservative *COX1* gene of the parasite led to identification of five different haplotypes in the research area. Two of the haplotypes were previously recorded internationally, while three new haplotypes associated with five novel mutations were recorded for the first time in the study region.

**Conclusion:**

A phylogenetic analysis revealed a strong relationship among *O. ovis* populations from various countries. The current research offered valuable molecular data for *O. ovis* species, essential for evaluating phylogenetic relationships and identifying these parasites at a molecular level.

## Introduction

Oestrosis is a condition of nasal myiasis caused by infestation from the larvae of flies in the *Oestrus* genus (Diptera: Oestridae). It is considered a serious parasitic disease in goats and sheep as well as other animal species at times (20). According to Bosly (10), the most frequent clinical signs in animals are sneezing and nasal discharge. Parasitic larvae significantly impact the host’s health and result in decreased production of milk, meat and wool (4). The sheep nose botfly (*Oestrus ovis* L.) induces serious cavitary myiasis in regions globally where goats and sheep are raised (14). *Oestrus ovis* can be identified by examining the morphological characteristics of the larvae, such as the slits on the posterior spiracular plates, by noting the clinical signs, and sometimes by finding adult flies (30).

A few years ago, molecular methods were widely utilised to gain understanding of the classification and categorisation of different insects. In particular, certain regions of ribosomal and mitochondrial DNA have been observed to be strong genetic indicators for addressing taxonomic identification challenges in certain myiasiscausing flies from the Oestridae family, as discussed in studies by Otranto *et al*. (31, 33) and Otranto and Traversa (32). The *mtCOX1* gene was selected to identify the larval relationships within the Oestridae family. Because of its large size and the presence of both highly conserved and variable regions with varying rates of mutation, it has been shown to be essential for different molecular phylogenetic purposes (25). The *COX1* 658 base pair (bp) area is a common and widely accepted genetic marker for animal taxonomy (19). Over the past decade, DNA barcoding has proven to be an effective method for examining genetic relationships and distinguishing various insect species in the Oestridae family using *COX1* sequences (24, 28).

Several research studies have been carried out on *O. ovis* in the northern cities of Iraq (21, 39), as well as in specific Arabic countries (1, 26, 29). Iran (40), Türkiye (5, 34), and other neighbouring countries have also been taken into account. Infestation with *O. ovis* in sheep is found in varying percentages across Europe, from 21.9% to 93.7% (8). Dorchies *et al*. (13) reported a rate of 28.4% in France, while Alcaide *et al*. (2) stated that in Spain, the rate was 71.1%.

The existing research on *O. ovis* occurrence and molecular characteristics in the Kurdistan region of Iraq, particularly in Sulaymaniyah province, is limited. The goals of this research were to evaluate the monthly and seasonal presence of *O. ovis* and associated risk factors in slaughtered sheep at a Sulaymaniyah abattoir, and to investigate the genetic relationships of *O. ovis* larvae using PCR and partial sequencing of the *mtCOXI* gene.

## Material and Methods

### Research area and parasitological analysis

During the period of September 2023 to August 2024, a total of 328 sheep heads were examined for *O. ovis* larvae in the nasal cavity and paranasal sinus at the Modern Sulaymaniyah abattoir in Sulaymaniyah province, Kurdistan Region, north-east Iraq. The sheep included 158 males and 170 females and 142 young and 186 adult animals, and comprised 128 individuals of the local breed and 200 examples of imported breeds. The region’s coordinates span from 35°04' to 36°30′ latitude and 44°50′ to 46°16′ longitude. Seasonal precipitation occurs in the area from October to May. The farmers in this area lack proper understanding of how to care for ruminants with external parasites and manage the infestations.

The larvae obtained were quantified, cleaned with physiological saline, fixed in 10% formalin and categorised by their developmental stage using Zumpt’s (48) descriptions.

### Molecular biology analysis

DNA was obtained from each of the *O. ovis* larvae gathered utilising a commercial DNA extraction kit (EasyPure Genomic DNA Kit, TransGen Biotech, Beijing, China) in accordance with the manufacturer’s instructions. A Genova Nano spectrophotometer (Jenway, Stone, UK) was utilised to measure the concentration of the extracted DNA that was pure.

A FFCOI forward (5′-GGAGCATTAATYG-GRGAYG-3′) and R-HCO reverse (5′-TAAACTTCAG GGTGACCAAAAATCA-3′) primer pair was used to amplify the mitochondrial *COX1* gene region (9, 15). The gene was amplified by PCR using the proofreading enzyme *f-Pfu* DNA polymerase from SBS Genetech (Beijing, China) following the conditions outlined by Rajabi *et al*. (38). The amplicons were confirmed through gel electrophoresis on 1.5% agarose gels and dyed with GoodView Nucleic Acid Stain (SBS Genetech).

Out of a total of 73 amplicons, 30 were purified and sequenced with the use of an upstream primer. DNA fragments extracted from agarose gel were purified using the SiMax PCR Products/Agarose Gel Purification Kit (SBS Genetech). Macrogen (Seoul, South Korea) partially sequenced all purified DNA using the Sanger method.

The ClustalW algorithm was employed for editing and aligning the gene sequences (45). After that, all sequences were submitted to the National Center for Biotechnology Information (NCBI) GenBank database and given accession Nos PQ218938–PQ218942.

The basic local alignment search tool (BLAST) algorithms were utilised to find similarities between the *COX1* gene sequence of *O. ovis* larvae from this research and other sequences in the GenBank database. A phylogenetic tree was constructed by comparing and aligning the coding genes in the *O. ovis* mitochondrial genome with reference sequences of other *O. ovis* and *Chrysomya bezziana* (as an out-group) using the neighbour-joining method (Table 1). The genetic distances were determined utilising Kimura’s two parameter model and the reliability of the tree structure was evaluated through the bootstrap value of 1,000 replicates of the datasets present in MEGA 11 (16, 43).

**Table 1.** The global GenBank accession numbers of *O. ovis* and *Chrysomya bezziana* utilised for multiple sequence alignment and phylogeny

Parasite	GenBank Accession No.	Origin	Host	Reference
	MW555828 MW555829	Iraq	Human	Jabir and Alomashi, 2021 unpublished
	MZ973002	Iran	Sheep	Rajabi *et al*. (38)
	MW316742	Iran	Human	Keshavarz *et al*. (23)
	KX268655	Iran	Sheep bot fly	Pape *et al*. (37)
*Oestrus ovis*	MT124626	Türkiye	Human	Karademir *et al*., 2020 unpublished
	MN845130	Croatia	Human	Pupic-Bakrac *et al*., 2019 unpublished
	MG755264	Bosnia and Herzegovina	Human	Pejic *et al*., 2018 unpublished
	ON000070	India	Sheep	Nagarajan *et al*., 2022 unpublished
	KR820703	Brazil	-	Marihno *et al*., 2016 unpublished
	OR440699	Mexico	Human	Olivera-Perez *et al*., 2023 unpublished
	NC_059851	USA	-	Aleix-Mata *et al*., 2023 unpublished
*Chrysomya bezziana*	MT787554	India	Human	Meghana *et al*., 2020 unpublished

### Statistical analysis

The Chi-square (χ2) test was utilised to analyse the data. Significant statistical findings were those with P-values less than 0.05.

## Results

### Rate of infestation

Out of the 328 sheep heads inspected, 73 (22.25%) were found to be infected with *O. ovis*. Table 2 provides information on the seasonal and monthly trends in both the prevalence and intensity of infection. Summer had the highest percentage of seasonal prevalence in sheep (34.09%), while winter had the lowest (10.95%). Significant variations were observed among the different seasons of the year (P-value < 0.05).

**Table 2.** Monthly and seasonal prevalence variation of *Oestrus ovis* and its larval stages infestation in slaughtered sheep in Sulaymaniyah province, Iraq

Month	Inf./n	(%)	MI	L1	L2	L3	TLC	Season Inf./n (%)	*X^2^* (P-value)
September/2023	5/29	17.24	3.20	3	5	8	16	Autumn 15/79 (18.98)	13.00 (<0.05)
October/2023	7/26	26.92	3.57	6	5	14	25		
November/2023	3/24	12.00	4.00	0	3	9	12		
December/2023	2/23	8.69	5.00	0	6	4	10	Winter 8/73 (10.95)	
January/2024	1/25	4.00	7.00	0	3	4	7		
February/2024	5/25	20.00	3.60	1	10	7	18		
March/2024	9/30	30.00	1.44	1	5	7	13	Spring 20/88 (22.72)	
April/2024	4/29	13.79	6.00	0	9	15	24		
May/2024	7/29	24.13	2.71	2	4	13	19		
June/2024	14/31	45.16	3.21	9	8	28	45	Summer 30/88 (34.09)	
July/2024	10/30	33.33	3.30	9	11	13	33		
August/2024	6/27	22.22	4.00	6	6	12	24		
Total (%)	73/328	22.25	3.36	37 15%	75 30%	134 54%	246		

1 Inf./n – number of heads infested/number of heads examined; MI – mean intensity; L1, L2 and L3 – larvae in stages 1, 2 or 3; TLC – total larvae count

For infected animals, the average number of larvae seen per animal each month varied from 1.44 to 7, with an average infection intensity of 3.36 larvae. The infestation rate peaked in June (45.16%) and hit its lowest point in January (4%). The three larval stages were seen all year round, except for the absence of first-stage larvae in January, April, November and December. There was a significant decline in the percentage of first stage larvae (L1) during winter. Out of the total of 246 larvae collected, 15% were in the L1 stage (37 larvae), 30% were in the L2 stage (75 larvae), and 54% were in the L3 stage (134 larvae). The peak amount of third instar larvae (39.55%) was seen in the summer, and the highest percentage of first instar larvae also occurred during that time (64.86%).

The summer had the highest number of larvae (102), followed by spring with 56 larvae, autumn with 53 larvae and winter with 35 larvae.

### Risk factors

To further understand the epidemiology of the infestation in the study area, the risk factors related to the oestrosis were investigated. For this purpose, the rate of infestation was studied according to the gender, age and breed (Table 3).

**Table 3.** Risk factors contributing to the prevalence of *Oestrus ovis* infestation in slaughtered sheep in Sulaymaniyah province, Iraq

Overall	Number of heads examined 328	Number (%) of heads infested 73 (22.25)	*X^2^* (P-value)
Sex			
Male	158	27 (17.08)	4.70 (< 0.05)
Female	170	46 (27.05)	
Age			
Young (≤1year)	142	23 (16.19)	5.31 (< 0.05)
Adult (> 1year)	186	50 (26.88)	
Breed			
Local breed	128	20 (15.65)	5.33 (< 0.05)
Imported breed	200	53 (26.50)	

It was found that the sex, age and breed of sheep had a significant effect on the risk of infestation. The rate of infestation was significantly higher in female sheep (27.05%) than in males (17.08%; P-value < 0.05). Also, the rate of infestation was significantly higher in adult sheep (>1 yr; 26.88%) than in sheep that were less than or equal to one year old (≤1 yr; 16.19%; P-value < 0.05). In addition, the rate was significantly higher in imported breeds (26.50%) than in local breeds (15.65%; P-value < 0.05).

### Gene sequence analysis

**Fig. 1.**
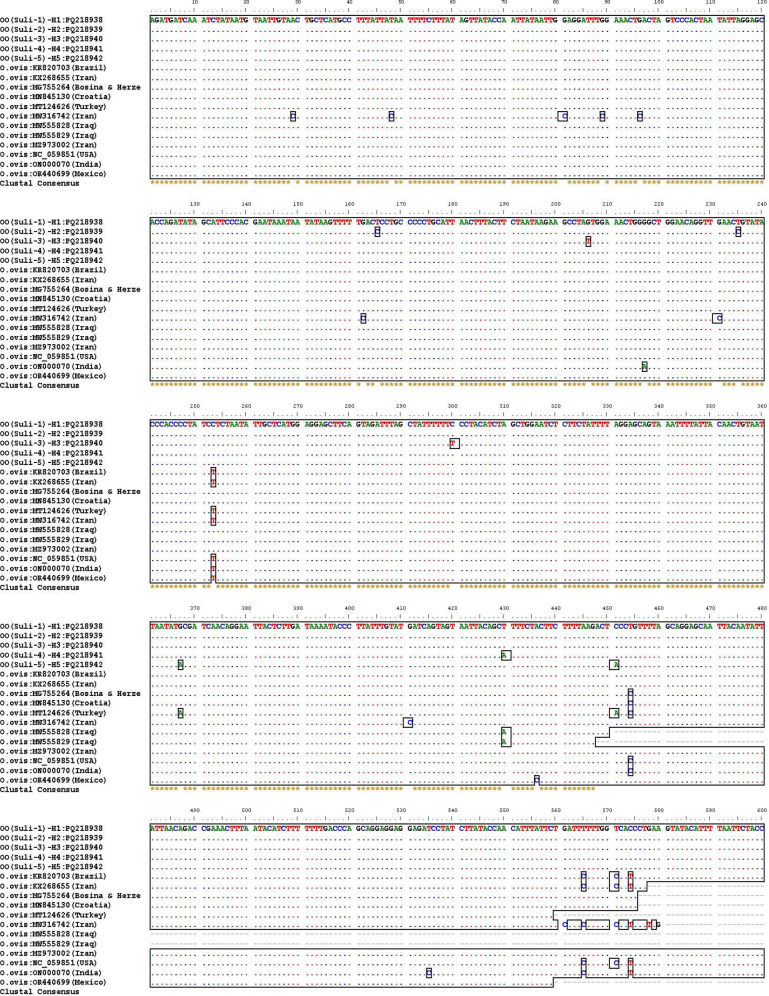
Multiple partial mitochondrial COX1 gene sequence alignments of *Oestrus ovis* in sheep isolates in Sulaymaniyah, Iraq. Nucleotide sequences recorded in the current study were labelled as OO(Suli-1)-H1–OO(Suli-5)-H5 with accession Nos PQ218938 – PQ218942

After editing and trimming the studied sequences, a PCR product with a length of 600 bp was achieved. The examination and matching of the sequences indicated that *O. ovis* had five distinct haplotypes, each with different mutations (Fig. 1). This study identified five haplotypes in the COX1 gene, designated as OO(Suli-1)-H1–OO(Suli-5)-H5 (Table 4).

**Table 4.** *Oestrus ovis* haplotype distribution patterns from sheep in Sulaymaniyah, Iraq, based on mitochondrial *COX1* gene sequences and accession numbers

*Oestrus ovis* haplotypes	Number of samples (isolates)	GenBank accession No.
OO(Suli-1)-H1	10	PQ218938
OO(Suli-2)-H2	8	PQ218939
OO(Suli-3)-H3	6	PQ218940
OO(Suli-4)-H4	4	PQ218941
OO(Suli-5)-H5	2	PQ218942

**Fig. 2.**
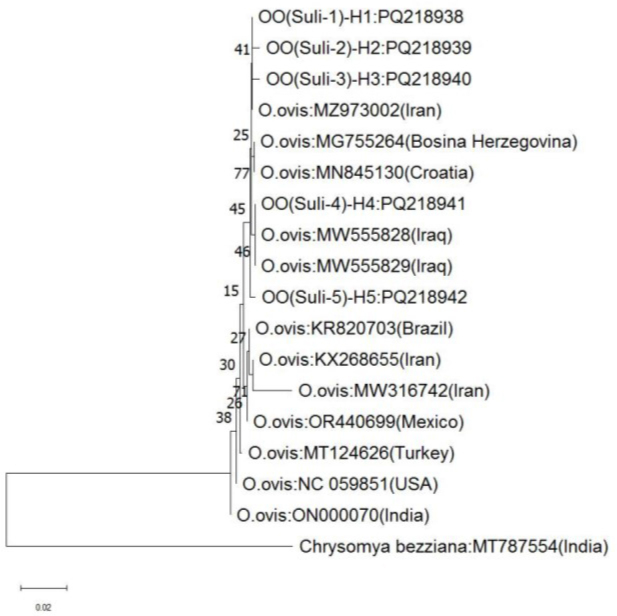
Phylogenetic tree for sheep isolates of *Oestrus ovis* in Sulaymaniyah, Iraq determined using the neighbour-joining method based on partial mitochondrial *COX1* gene sequences. The scale bar corresponds to a 2% variation in nucleotide sequences

The BLAST analysis revealed that all of the sequences shared a significant resemblance to the *O. ovis* “sheep botfly” in GenBank. All sequences that were analysed were found to be closely related to the Iranian isolate of *O. ovis* (GenBank accession No. MZ973002), showing identities ranging from 99.67 to 100%. According to the analysis of COX1 sequence data, all haplotypes of *O. ovis* were found within one branch of the phylogenetic tree (Fig. 2).

In the region, five distinct haplotypes were identified. Three of the haplotypes (OO(Suli-2)-H2, OO(Suli-3)-H3, OO(Suli-5)-H5) were found solely in the research area and included five novel mutations at positions: 165 (T > C), 206 (G > T), 235 (T > C), 300 (C > T), and 451(C > A). The other two haplotypes (OO(Suli-1)-H1, OO(Suli-4)-H4) were previously identified and were 100% identical to accession Nos MZ973002 in Iran, MW555828, and MW555829 in Iraq, respectively.

The nucleotide alignment results of PQ218938–PQ218942 with other sequences showed variations in the phylogenetic tree at specific positions: 29 (A > C) for MW316742, 48 (T > C) for MW316742, 81, 89, 96, and 162 (G > C) for MW316742, 165 (T > C) for PQ218939, 206 (G > T) for PQ218940, 217 (G > A) for ON000070, 231 (G > C) for MW316742, 235 (T > C) for PQ218938, 253 (C > T) for KR820703, KX268655, MT124626, MW316742, NC_059851, ON000070, and OR440699, 300 (C > T) for PQ218940, 367 (G > A) for PQ218942 and MT124626, 411 (G > C) for MW316742, 430 (T > A) for PQ218941, MW555828, and MW555829, and 436 (A > C) for OR440699 (Fig. 1).

## Discussion

In this study, the occurrence of *O. ovis* in sheep was found to be 22.25%, mirroring rates reported by Metwally *et al*. (26) in Saudi Arabia (22.3%), by Karatepe *et al*. (22) in Türkiye (22.52%) and by Shareef (39) in the Sulaymaniyah province of Iraq (20.96%). This discovery shows a lower rate compared to previous studies in different countries: 49.7% in Iran by Shoorijeh *et al*. (40), 38.71% in Türkiye by Özdal *et al*. (34), 58.03% in Jordan by Abo-Shehada *et al*. (1), 42.33% in Libya by Negm-Eldin *et al*. (29), 43.2% in Greece by Papadopoulos *et al*. (35), 71.1% in Spain by Alcaide *et al*. (2) and 91% in Italy by Caracappa *et al*. (12). On the other hand, certain authors documented lower occurrence rates: 8.67% in Egypt (3), 13.7% in Brazil (42), 17.2% in the Nineveh province of Iraq (21) and 24.8% in France (13). The diversity in prevalence rates might be due to variations in fly population, host immunity, animal types, weather patterns, farming techniques, medication usage and geographical location.

The average infestation intensity was 3.36 larvae per host, surpassing Shareef's (39) findings of 1.98 larvae per sheep in Iraq, but lower than Özdal *et al*.’s (34) results of 4.02 larvae per sheep in Türkiye. The variability in climate, age of animals and differences in host resistance could account for this (34).

The study revealed a greater proportion of L3 larvae (54%) as compared to L2 (30%) and L1 (15%). Yilma and Dorchies (47), Özdal *et al*. (34) and Mohammed *et al*. (27) reported a higher proportion of L3 compared to L2 and L1 in France, Türkiye and Iraq, respectively. Similarly, Alcaide *et al*. (2) found a higher percentage of L1 compared to L2 and L3 in south-western Spain, where the climate and altitude are similar. Larvae in the L1 stage may have been missed because of their small size and possible concealment in places like turbinates and ethmoid bones, or may have been destroyed in the nasal cavities during the hypobiotic phase (7).

The sheep had the highest infestation rates in summer and spring and the lowest in winter. In summer, the adult *O. ovis* fly becomes more active, as reported by Dorchies *et al*. (13), whereas in winter, the larvae experience periods of diapause and a reduction in their growth rate, as indicated by Yilma and Dorchies (47). This study showed that the rate of infestation in sheep was greatly impacted by the varying seasons. This is consistent with the investigation carried out by Özdal *et al*. (34) and differs from the research by Metwally *et al*. (26) that showed no significant effect of seasons on nasal botfly infestation levels. A study conducted in Egypt by Amin *et al*. (3) showed that infestation rates were highest during autumn at 17.91%, while winter had the lowest rate at 7.85%. Concordantly, researchers in Iraq found that the highest percentage was in autumn at 76.92%, while the lowest was in winter at 30.76% (27). Changes in environmental conditions could be the cause of these variations.

The occurrence of oestrosis varied from 4% in January to 45.16% in June. This aligns with findings from Özdal *et al*. (34), but contradicts Karatepe *et al*. (22) who found the peak infestation rate to be in October. This research demonstrated that all larval phases were observed consistently during the whole period, except for the absence of first-stage larvae in January, April, November, and December. Shareef (39) observed that Iraq only had second and third instar larvae present. Even though the overall presence of first stage larvae was low in the study, there was a higher prevalence of L1 larvae from June to October, possibly because of new larvae from adult flies. Larvae of *O. ovis* stop growing during hypobiosis, whether it be in winter in temperate regions or during the hot and dry season in Sahelian countries (47).

We examined how risk factors like gender, age and breed impacted the results. We discovered that the breed of sheep had a notable impact on the rate of infestation. Infection rates were notably more elevated in imported breeds compared to local breeds (26.50% *vs* 15.65%, P-value < 0.05). Possible explanations for this could involve animals’ adaptation to the surroundings, genetic differences, previous exposures and stress factors. A significantly higher occurrence of oestrosis was observed in female animals in comparison to male animals (27.05% *vs* 17.08%, P-value < 0.05). The findings align with the previous studies conducted by Uslu and Dik (46) and Shoorijeh *et al*. (40). On the other hand, Metwally *et al*. (26), Mohammed *et al*. (27) and Negm-Eldin *et al*. (29) observed a higher incidence of the condition in males as opposed to females. The higher number of female animals in the groups could lead to a higher susceptibility to *O. ovis* larva infestation, which is also influenced by differences in physiology and behaviours between males and females (29). Oestrosis is found in a higher percentage of adult sheep (26.88%) compared to sheep (16.19%) that are 1 year old or younger. This discovery is consistent with those of several research studies (1, 29, 35, 39, 46), but contrasts with those of other investigations (5, 22). One potential explanation for the higher prevalence in mature animals may be their heightened appeal to female flies and their larger nasal openings in comparison to juvenile animals. Furthermore, mature animals exhibit decreased respiratory rates compared to juvenile animals, potentially assisting the female fly in egg laying and larvae entering nasal sinuses. Moreover, a juvenile animal may possess antibodies against oestrosis inherited from its mother, as suggested by Silva *et al*. (41).

According to Ogo *et al*. (30), the physical attributes and morphological identification of the larvae should not be relied upon. Molecular identification of fly species causing myiasis can be considered a dependable substitute for morphological identification because of the challenges in identifying larvae at the genus level (18, 30). Bosly (11) discovered *O. ovis* larvae at a molecular level, demonstrating its potential as a dependable substitute for conventional morphological identification techniques. This study focused on investigating the genetic diversity of *O. ovis* by analysing *mtCOX1* gene sequences extracted from the nasal passages of sheep in Iraq at a molecular level. The mitochondrial DNA enables a more accurate identification of larvae species, with closely related species having unique developmental traits (17, 36).

In our study, the novel mutations resulted in new haplotypes that were previously not reported from any country. However, the sequences from our study had the highest similarity, ranging from 97.40 to 99.83%, with haplotypes isolated from Iraq (accession Nos MW555828–MW555829), Iran (accession Nos MW316742, KX268655), Turkey (accession No. MT124626), Croatia (accession No. MN845130), Bosnia and Herzegovina (accession No. MG755264), India (accession No. ON000070), Mexico (accession No. OR440699), Brazil (accession No. KR820703), and USA (accession No. NC_059851). These findings show that genetic differences may exist among samples collected from various regions. Hence, variations in *O. ovis* isolates might be due to differences in animal breeds, geographic locations, and climates (10, 29).

Phylogenetic analysis showed that the sheep sequences in this study had intraspecific variations and were highly similar to *mtCOX1* gene sequences of *O. ovis* isolates from sheep in various countries including Iraq, Iran, Türkiye, Croatia, Bosnia and Herzegovina, India, Mexico, Brazil and the USA. On the other hand, Rajabi *et al*. (38) found that there was no variation within isolated sequences from Iranian sheep. There is a higher level of diversity within the genes encoding proteins in mtDNA when compared to ribosomal genes, as observed by Tan *et al*. (44). The research suggests that similarities among Iraqi isolates, Iran and Türkiye may be due to common geographical conditions and increased trade, as indicated by molecular alignment and phylogenetic tree analysis (6). Moreover, all *O. ovis* samples analysed in this study, as well as those retrieved from GenBank, were clustered together in a monophyletic group, consistent with earlier research (10, 26, 29, 38).

## Conclusion

The current research indicates that oestrosis is a significant issue for sheep in Sulaymaniyah province in Iraq, and it is anticipated to impact animal production and welfare. The findings showed that the infestation rate was 22.25% in total and was notably higher in imported breeds compared to local breeds, adults compared to young sheep, females compared to males, and during the summer season compared to other seasons. The study area contained five unique haplotypes; two had been identified in Iraq and Iran before, while three were specific to our region. Five new mutations were discovered within regional haplotypes. This study provides valuable molecular information on *O. ovis* larvae in sheep in Iraq through the analysis of *COX1* sequence barcodes. The results indicated a strong correlation among *O. ovis* strains from various countries, despite the dataset including only a few sequences. The study findings demonstrate that *COX1* is an effective molecular marker for identifying and characterising botfly species.
